# Review of Microwave Near-Field Sensing and Imaging Devices in Medical Applications

**DOI:** 10.3390/s24144515

**Published:** 2024-07-12

**Authors:** Cristina Origlia, David O. Rodriguez-Duarte, Jorge A. Tobon Vasquez, Jean-Charles Bolomey, Francesca Vipiana

**Affiliations:** 1 Department of Electronics and Telecommunications, Politecnico di Torino, 10129 Torino, Italy; cristina.origlia@polito.it (C.O.); david.rodriguez@polito.it (D.O.R.-D.); jorge.tobon@polito.it (J.A.T.V.); 2 Université Paris-Saclay, 91190 Paris, France; jcbelex@orange.fr

**Keywords:** biological tissues, blood glucose monitoring, breast imaging, dielectric measurements, brain imaging, microwave sensing, image-guided intervention, knee injuries, medical imaging, microwave imaging, stroke diagnosis, thermal ablation, torso scanning

## Abstract

Microwaves can safely and non-destructively illuminate and penetrate dielectric materials, making them an attractive solution for various medical tasks, including detection, diagnosis, classification, and monitoring. Their inherent electromagnetic properties, portability, cost-effectiveness, and the growth in computing capabilities have encouraged the development of numerous microwave sensing and imaging systems in the medical field, with the potential to complement or even replace current gold-standard methods. This review aims to provide a comprehensive update on the latest advances in medical applications of microwaves, particularly focusing on the near-field ones working within the 1–15 GHz frequency range. It specifically examines significant strides in the development of clinical devices for brain stroke diagnosis and classification, breast cancer screening, and continuous blood glucose monitoring. The technical implementation and algorithmic aspects of prototypes and devices are discussed in detail, including the transceiver systems, radiating elements (such as antennas and sensors), and the imaging algorithms. Additionally, it provides an overview of other promising cutting-edge microwave medical applications, such as knee injuries and colon polyps detection, torso scanning and image-based monitoring of thermal therapy intervention. Finally, the review discusses the challenges of achieving clinical engagement with microwave-based technologies and explores future perspectives.

## 1. Introduction

Microwave (MW) imaging and sensing entered the medical domain in the mid-1980s as evidenced by the body of work presented by Larsen and Jacobi in [1]. Initially, the primary focus was on non-invasive microwave dosimetry in biosystems. However, researchers soon realized the broader potential for progress in diagnostic and therapeutic medicine by harnessing the appealing advantages of MWs. These advantages include their non-ionizing nature, effective tissue penetration capabilities, and sensitivity to dielectric contrasts within the human body based on tissue type and specific pathological conditions.

The era of “clinical acceptance” began to take shape in the early 2000s [2], marked by the development of initial equipment prototypes for selected applications on human subjects, driven by the advancements in computing power and image reconstruction algorithms. Additionally, the reduced cost and complexity of hardware components have facilitated the miniaturization and integration into portable, wearable devices with networking capabilities. A wide range of applications has been explored, typically working across the frequency range from 1 GHz to 15 GHz, encompassing various biosensors for ex vivo dielectric characterization of human cells or vital sign detection [3], also integrated in wearable sensing systems [4], skin tumor diagnosis [5], and blood glucose monitoring [6]. Image-based diagnosis and monitoring have been explored for breast cancer [7], axillary lymph nodes [8], brain stroke [9], knee injuries [10], and thoracic diseases [11].

The collaboration between academia and industry has yielded significant milestones in the development of operational MW imaging systems for breast screening and brain stroke diagnosis and monitoring. The keen interest in these applications is driven by the need to support the conventional clinical tools when encountering inherent constraints, such as the use of harmful X-rays in mammography or brain computed tomography (CT), as well as the high costs and bulky non-portable equipment for magnetic resonance imaging (MRI) diagnosis. Moreover, these technologies require long examination times and are not always available, especially in developing countries or rural areas. Notable reports include a comprehensive review of breast screening clinical studies published in [12] (2022) and an assessment of functional brain stroke scanners in [9] (2023), which reviews the functional brain stroke detectors, highlighting recent validations on volunteers and patients. While promising outcomes have emerged from these trials, aligning with preliminary numerical studies and phantom tests, challenges remain due to inconsistencies in the outcome descriptions (e.g., image quality metrics, clinical effectiveness assessment, and differences in the trial populations). This heterogeneity hinders a fair comparison and a complete understanding of the actual potential of these systems [12].

The path to establishing microwave (MW) technology in clinical settings has been significantly longer compared to traditional imaging and sensing modalities due to several factors. A critical concern for diagnostic imaging is maintaining adequate spatial resolution for the intended application while ensuring sufficient tissue penetration. Generally, higher frequencies offer better spatial resolution but at the cost of increased attenuation, resulting in reduced penetration depth. Typically, the operational frequency for a given application is chosen as a compromise between these two factors. For example, breast and brain imaging have optimal working bands around 3 GHz and 1 GHz, respectively, providing resolution in the order of centimeters. However, as noted in [2], additional factors can enhance spatial resolution, such as multi-frequency and multi-view data acquisition approaches and the use of an appropriate matching medium surrounding the sensing probes. Another significant challenge lies in understanding and modeling the complex interaction between tissues and electromagnetic (EM) radiation, usually addressed using simplified models due to computational limitations. Despite the wealth of experimental data available on tissue dielectric properties, recent works emphasize the crucial need for a unified database with a standardized measurement procedure [13,14]. Nonetheless, unavoidable modeling errors stemming from inter-individual variability significantly impact performance, particularly in MW medical imaging.

Medical MW imaging applications exploit differences in the dielectric properties of human tissues, specifically between healthy and diseased tissues, to produce distinct responses to EM radiation. The sensing system, consisting in a set of transmitting and receiving probes surrounding the body, investigates the domain of imaging (DoI) by emitting an incident electric field and capturing the resulting altered field. An inversion algorithm then processes the measured data to reconstruct a dielectric profile of the scattering object. Two primary challenges affect the inversion process: first, the non-linearity derived from multiple scattering effects and the intrinsic relationship between the total internal field and the dielectric contrast; second, the ill-posedness due to the compactness of the forward scattering phenomenon, necessitating the use of some regularization schemes to stabilize the solution. Common image reconstruction algorithms typically rely on pre-computed EM models of the sensing scenario and use measured data in the form of frequency-domain scattering signals or their time-domain transformations. The literature presents various imaging strategies that can be categorized into three main groups. On the one hand, there are direct inversion methods, based on linear models that neglect multiple scattering phenomena within the DoI, often leveraging well-known approximations such as the Born or Rytov approximations [15]. These methods reduce complexity and non-linearity, which is advantageous for quickly retrieving qualitative information about the shape and location of a scatterer (i.e., an area of dielectric contrast compared to the reference scenario). Similarly, radar-based methods can only identify and locate strong scatterers inside the object under study and are commonly used in operational scanners addressing real-time reconstruction [16]. Lastly, quantitative tomography is chosen when knowing the dielectric properties value is crucial, such as for tissue type differentiation. Here, non-linear iterative inversion procedures are employed relying on accurate EM modeling, which requires greater computational resources and time. These methods address challenges associated with the problem’s ill-posed nature, including issues like local minima solutions, increased sensitivity to modeling errors, and dependence on the initial guess in the iteration process. Integration of prior knowledge about the dielectric profile of the DoI into the imaging process has been shown to enhance performance and effectiveness in clinical deployment [17]. It is worth noting that there is not a one-size-fits-all efficient imaging technique; the choice depends on the experimental design, final application, available computational capability, and targeted costs.

**Figure 1:**
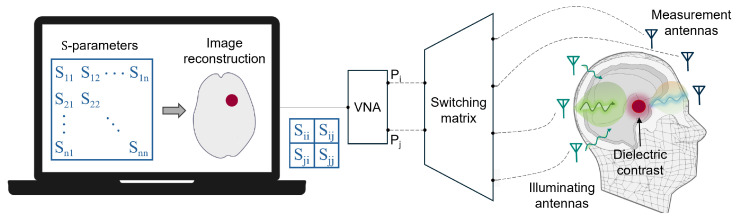
General scheme of an electronically scanned multi-probe system for brain imaging.

In terms of hardware design, the transceiver component is realized, aiming to maximize the sensitivity and dynamic range, which is crucial for detecting weak biological signals embedded in considerable environmental noise. Typically, a Vector Network Analyzer (VNA) is employed to generate and gather MW signals and interfaces with mechanically scanned setups (that use a low number of antennas physically moved for spatial sampling) or electronically scanned multi-probe arrays. In the first case, a key point is the trade off between a fast scanning and the accuracy in the antenna positioning. In the second case, the scanning is performed with electro-mechanical or (faster) solid-state RF switches [18]; we do not have any antenna movement issue, but there are limitations in terms of switch insertion loss, isolation, repeatability, and non-linearity. Recently, to overcome such limitations in the RF frequency bands, in [19], intermediate frequency (IF) switching for efficient multiplexing with several sensors has been proposed. The diagram in Figure 1 outlines a general electronically scanned multi-probe system applied for brain imaging, where a control unit collects the scattering parameters data (*S*) for each antenna pair (*i*,*j*) to retrieve the image.

The design of antennas plays a crucial role in determining the detection capability of MW medical imaging and it is directly related to practical operating parameters such as frequency bandwidth, near-field radiation, manufacturing complexity, and costs. As MW medical imaging devices mostly work in the near-field, further complex considerations are involved. The literature review in [20] reports the antennas employed for inspecting different body areas and pathologies. The authors highlight that working at lower frequencies is preferable for imaging bulky tissues due to lower attenuation. Moreover, image resolution can be enhanced by working in a larger frequency bandwidth, as well as using more antennas. However, it is acknowledged that spatial resolution is ultimately governed by the available signal-to-noise ratio (SNR); thus, increasing the number of sensors implies that a larger dynamic range is required [2,21]. These considerations underscore the complex balance required in antenna design to optimize performance while managing practical constraints.

**Figure 2:**
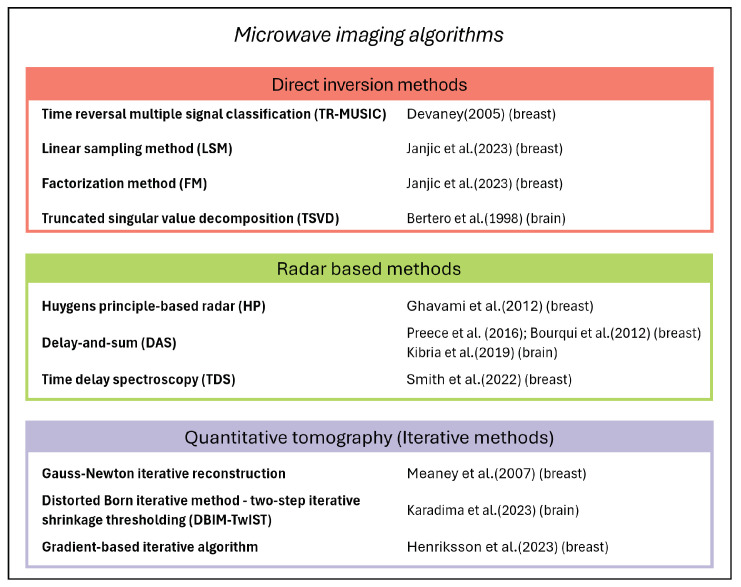
Scheme depicting classes of MW imaging: direct inversion methods, radar-based methods and quantitative tomography. The listed algorithms are common strategies applied in advanced systems for breast and brain imaging and are reported together with the cited papers: Devaney [22] (2005), Janjic et al. [23] (2023), Bertero et al. [24] (1998), Ghavami et al. [25] (2012), Preece et al. [26] (2016), Bourqui et al. [27] (2012), Kibria et al. [28] (2019), Smith et al. [29] (2022), Meaney et al. [30] (2007), Karadima et al. [31] (2023), Henriksson et al. [32] (2023).

This work aims to provide an overview of some of the most promising biomedical applications of MW sensing and imaging. The goal is to discuss and compare various systems settings and their effectiveness, presenting the latest achievements in the field, with a focus on major results from clinical trials and promising technological trends. The selected applications are characterized by near-field operation in the frequency band 1–15 GHz, typically exploited for tissue inspection ranging from large areas of the body to the more localized functions (e.g., blood sensing just beneath the skin surface). In Section 2, we analyze current insights into human tissue dielectric properties. This section focuses on reviewing the latest guidelines established by the research community for measuring and reporting consistent data, and identifying existing reference databases. Section 3 and Section 4 are dedicated to breast and brain imaging applications, respectively. The most advanced devices are reviewed and compared based on the technical behavior and capabilities demonstrated in clinical trials on patients. In order to provide the reader with additional guidance, Figure 2 classifies the main imaging algorithms cited in these sections.

Furthermore, blood glucose sensing, one of the most promising applications that has not yet entered the market, is discussed in Section 5. Finally, the remaining cutting-edge implementations of MW medical imaging are collected in Section 6. Conclusions and perspectives of MW imaging and sensing in the medical field are drawn in Section 7.

## 2. Dielectric Characterization of Human Tissues

Biological tissues expose a high variability of dielectric properties (DPs) at MW frequencies, meaning that their response to EM waves is different depending on the tissue type. When an electric field E is applied to a dielectric material, it induces a total displacement flux D:

(1) D=εE,

assuming an isotropic material, where ε is the complex permittivity characterizing the material, whose real part refers to the capability to store energy, while the imaginary part relates to the losses, which comprehends conductivity losses. The main factor determining the dielectric dispersion characteristic (i.e., their dependence from frequency) is the water content [33] such that drier tissues, e.g., skull and fat, exhibit a lower variation in frequency of their permittivity, while tissues with more water such as muscle, brain, and blood have a high one. Diagnostic imaging aims to detect the DPs changes induced in the pathological tissue, which in tumors is related to the increase in water molecules compared to the healthy surrounding area. Since the early 1980s, many ex vivo dielectric studies have investigated soft tissue tumors affecting, among others, breast, colon, kidney, liver, spleen, lung, muscle and blood vessels [34,35,36], suggesting the feasibility of MW detection, even if, as noted in [16], results were not always quantitatively consistent and the measurement methodology did not take into account the complexity of the heterogeneous tissue. A case in point is later research on breast tissues, underlying that normal DPs encompass a broad range of values, depending on adipose and fibro-glandular tissue content, and only the contrast between malignant and adipose-dominated healthy tissue is large, while it significantly decreases with high fibroglandular densities [37,38].

The first dielectric investigations of biological tissues came in parallel with the arrival of mobile phones and was initially required for dosimetry and evaluation of the influence of EM exposure in humans. Nowadays, recent advances in biomedical MW techniques need accurate knowledge of EM characteristics for building reliable physical and numerical testing models, necessary to design and validate new MW imaging and sensing devices.

Various techniques exist to measure the dielectric properties of biological tissues. These include transmission line, open-ended coaxial probe, and image-based techniques [39]. Among different methods, the open-ended coaxial probe is the most common one to measure tissue properties in the operational frequency range of most MW applications [40]. It is a non-destructive technique, easily applicable both in vivo and ex vivo, with relatively simple preparation of the tissue sample. Common practices of coaxial probe measurement were reviewed and discussed in [40], where the authors addressed, among others, the following topics:

- Description of the standard calibration procedure and related confounders (e.g., environmental variables, VNA drift, and cable movement);
- The validation procedure with a known reference liquid;
- The uncertainty evaluation, usually based on the guidelines of the National Institute of Standard and Technology (NIST) [41];
- A summary of the comparative studies with both in vivo and ex vivo measurements;
- The best practice measurement steps, with special considerations about the sensing depth, the tissue heterogeneity, and the effect of temperature on tissue DPs.

Characterizing heterogeneous biological samples with a coaxial probe is an open challenge, which consists in sensing each tissue component separately with high accuracy: [42] revealed that the radius and depth of a coaxial probe depend on the permittivity contrast between different tissue components and are strictly related to the portion of tissue closest to the inner conductor of the probe, finally demonstrating the possibility of numerically modeling the sensing radius in different scenarios. A comprehensive summary of the DPs derived in the literature for several types of tissue and malignant tumors is extracted in [39], starting from the first review by Gabriel et al. (1996) [43], up to the recent achievements in image-based dielectric estimation.

As emerged in many recent studies, there are two main reference databases available online for body tissue DPs, the IFAC-CNR database [44] and IT’IS Foundation database [45], both derived from Gabriel’s parametric models extracted from the first available collection of measured tissue properties, dating back to 1996 [46,47]. However, as pointed out in [39], later dielectric surveys only partially agreed with Gabriel’s modeling equations, depending on the tissue and the frequency; principal inconsistencies were found for adipose tissue, which may be explained by the variability of fat and water content in the sample. The anisotropy of tissues, mostly studied for muscles, is also a key factor determining the EM response and thus should be taken into account in future studies. Regarding the malignant tissues, it was reported that there is a lack of data measured on real human tissues, a primary issue to be addressed considering the high relevance for ongoing research on diagnostic and monitoring applications. Furthermore, from the analysis in [39], we can observe systematic variations between in vivo and ex vivo data, ascribed from the majority of the literature to dehydration, blocked blood perfusion, and temperature changes in the excised tissue. At MW frequencies, the matter response is governed by polar molecules oscillations [14]; thus, the water content is a well-established measurement confounder, and generally the time between the excision and measurement, as well as the temperature and environmental condition influence the hydration of the tissue [14,48,49,50]. The effect of dehydration has been highlighted in [50] on porcine liver samples, reporting a variation of 9% in permittivity after 35 min at a physiological temperature, non-negligible in MW sensing applications; the authors suggested that predictive models should be investigated to compensate for the effect of environmental variables, together with precautions in tissue handling, such as air flow reduction.

As it is clear from the above, despite the abundance of literature, the comparison across studies is not straightforward due to the variability of measurement conditions and parameters, or even the lack of this information in reports. Recently, some groups put effort in this regard, in drafting standard guidelines for regularizing DP measurement procedures and data reporting.

The minimum information model for dielectric measurements of biological tissues (MINDER) was defined in [13] in 2017 and includes essential confounders to account for and metadata to be recorded to enable the analysis, comparison, and replication of published data. Recently, a working team in the COST MyWAVE network published a complete guideline with best-practices recommendations, that aims to standardize this branch of research [14]. The authors pointed out major confounders that should be compensated for in the measurements process, discussing in detail the role of tissue hydration, temperature of the calibration load, and sample size. The work tracked the following steps in the dielectric study process:

- Measurement setup (e.g., effects of cable movements on VNA settings, and probe use);
- Calibration;
- Sample characteristics;
- Measurement practices (e.g., repeating the measure several times is highly recommended);
- Data analysis, which comprises data fitting with mathematical equations and uncertainty calculation (according to GUM document by the Joint Committee for Guides in Metrology [51]);
- Data reporting methods, which satisfy EU directives, such as the FAIR guiding principles [52], and promote open-access data collections.

As a starting point, to encourage the adoption of common guidelines in this branch of research, ref. [14] proposes a custom data analysis software for automated filtering, modeling (e.g., by means of Cole–Cole or Debye models [33]), and uncertainty calculation, together with an open-source archive of dielectric and thermal properties, available online at [53], where researchers can contribute, following a guided process for data loading.

Considering the challenges that have emerged from the state-of-the-art review, alternative techniques for human tissues DPs estimation may be considered; in particular, the latest advancements in image-based methods have proven promising results, being intrinsically suitable for in vivo employment. Some relevant methods are summarized in [39], which are tomographic approaches based on electrical properties (EPT), electrical impedance (EIT), or magnetic resonance electrical impedance (MREIT), and imaging methods combined with artificial intelligence.

## 3. Breast Cancer Detection

Breast cancer stands as the most widespread malignancy among women worldwide. According to the latest updates from the World Health Organization (WHO), 2.3 million women received a diagnosis, with 685,000 deaths in 2020. Since the 1990s, survival rates have improved due to the spreading of national screening programs, allowing earlier diagnosis and effective medical therapies. Nevertheless, the WHO persists in its commitment to reducing cancer mortality, cognizant that the gold standard mammographic screening remains unfeasible in numerous countries [54].

### 3.1. Traditional Breast Imaging Techniques

The principal technologies in routine care are exhaustively discussed and compared in the literature [55,56]; the following summarizes the main features:

- Mammography employs potentially harmful X-rays, so frequent repeats of the procedure are not recommended, while breast compression-induced pain in some cases discourages women from attending screening programs (involving subjects aged > 40). Among the unresolved issues, there are the low sensitivity (<70%) attested in high-density breasts (usually younger aged women) with respect to 90% achieved in fat-dominated tissues, and high-rate of false-positive recalls. Digital breast tomosynthesis (DBT) partially solves the difficulties related to tissue overlap in dense breasts by collecting 3-D multiple projections, but this implies additional radiation exposure. Nevertheless, mammography provides high spatial resolution, and the performance decreases only when the lesion size is smaller than 20 mm [57].
- Breast sonography, or ultrasound (US), exploits the acoustic impedance of soft tissues, and it is sensitive to differences in fat, fibrous, and glandular components of the breast. It offers a non-ionizing, low-cost tool for the investigation of symptomatic cases and examination of dense breasts complementary to mammography, as well as real-time image-based guidance during needle biopsy. Nevertheless, its role as a screening method is debated due to its dependence on operator skill, long-time requirements, and higher rates of false positives [58]. While recent advancements in US technology have improved the achievable resolution and allow to automate the procedure, its adoption in clinical practice remains to be fully established.
- MRI creates detailed images of soft tissues based on the relaxation properties of the hydrogen atoms in the presence of a strong magnetic field and usually requires a contrast agent. It is applied for the high-resolution assessment of diagnosed breast lesions, for example, before surgery or to evaluate treatment response [55]. The outcomes of recent tests in screening populations foster the choice of this technique for high-risk patients, having higher sensitivity in finding neoplasms. However, its use is constrained by high costs, long acquisition times, limited portability and availability, together with the remaining doubts related to the high number of false detections [56].

### 3.2. Current Achievements and Drawbacks in Microwave Breast Imaging

MW imaging is proposed as an innovative modality within this context, being non-ionizing and non-invasive, rapid, and cost-effective. Most current operational systems do not require breast compression and have received favorable opinions regarding comfort and ease of use. Nowadays, breast cancer screening and detection are recognized as the most promising applications of MW imaging, where technological advantages perfectly fit unsolved clinical needs. Nevertheless, after more than two decades of research efforts, the difficult clinical acceptance inevitably poses questions about limitations and shortcomings in the actual scientific understanding as demonstrated by newest interest in reviewing various aspects of the state of the art [7,12,59,60,61,62].

The experimental dielectric characterization of breast tissues is documented in many works, starting from 1984, and chronologically described in recent reviews [59,60]. Despite the acknowledged methodological limitations of some studies and variability among the published results, some relevant outcomes have been confirmed supporting the feasibility of MW detection of cancerous breast masses. The first large-scale study from Lazebnik et al. [37] derived the Cole–Cole model in the frequency range 0.5–20 GHz and estimated a contrast of 10:1 between malignant and normal adipose-dominated tissues, which decreases at 10% between malignant and normal glandular/fibroconnective tissues. The largest measurement set collected in [63], with 509 samples data in the frequency range 0.5–8 GHz, confirmed previous results and significantly higher properties of benign tumor tissue with respect to normal breast tissues, noting that the optimal frequency maximizing MW sensitivity could be around 2.5 GHz, where the standard deviation of the parameters is lower. In 2023, Canicattì et al. [64] validated a custom setup method based on open-ended coaxial probe measurements up to 9 GHz, feasible for clinical analysis of excited tissues immediately after biopsy, and provided dielectric values for cancer, fibroglandular, and adipose tissue assessed with the histological gold standard. Recent advancements suggest the use of magnetic nanoparticles to selectively improve dielectric contrast for pathological tissues, promising to enhance the specificity of MW imaging [65].

The review in [7] chronologically summarizes the main findings in MW breast imaging research. It encompasses simulation-based studies on phantom and clinical trials, comparing image reconstruction methods and antennas, and offering a comprehensive overview of progress and prospects in the field. Antenna sensors have a key role in MW imaging systems, and recent advancements offer a wide choice in ultra-wideband (UWB) antenna technology, characterized by high-speed data acquisition, low interference, and cost-effectiveness [66]. The array geometry fits the breast shape, with the difficulty to adapt at different breast sizes keeping good matching at the skin–antenna interface. The capability to cover the entire breast volume, from the chest wall to the nipple with no shadowed zones, is highly desirable. One possibility is using syntheticarrays, where a low number of transmitters and receivers are mechanically moved to complete the surface scan, which usually implies longer scan times [61]. On the other hand, hardware arrays, where the antennas are fixed, are faster and avoid possible noise and artifacts due to antenna movements, but their design depends on the antenna size and mutual coupling effects. The use of a coupling medium (CM) is recommended to avoid strong reflections at the skin interface, but its employment is a non-trivial decision in the design process, especially with liquid CM, which also affects patients’ comfort.

In [12], Porter and O’Loughlin investigate the qualitative and quantitative metrics employed to evaluate image quality, clinical effectiveness, and efficacy, aiming to lead the way in standard guidelines and universally agreed definitions. The authors assert that common image-based metrics should be integrated with objective parameters not dependent on imaging resolution or the specific system. Among the most common parameters to measure the accuracy of diagnostic tests, sensitivity is the percentage of patients correctly identified as having a tumor, while specificity is the proportion of tumor-free cases accurately identified. It is noted that screening applications should target high sensitivity, while an efficient diagnostic device should maximize its specificity, where the two parameters usually have opposite trends. This relationship is represented through the area under the receiver operating characteristic curve (AUC), ranging between 0 and 1 (ideal case). Furthermore, many works evaluate diagnostic accuracy, but its definition is not unique and depends on the context. Regarding the trial populations, ref. [12] underlines the importance of homogeneity in the number of patients and their medical condition for a fair evaluation; for example, the specificity can be effectively assessed only when the asymptomatic population is properly represented, as expected in a real-world screening campaign. Reimer and Pistorius in [62] expand the analysis of the methodologies for the evaluation of the diagnostic performance with a scoping review which includes machine learning (ML)-based diagnostic applications and critical inspection of a variety of image quality metrics. The authors claim that overall image quality is determined by factors such as spatial resolution, contrast, contrast resolution, noise levels, accuracy, and artifacts, all of which should be assessed by standard metrics. For greater robustness, it is advisable to appraise the overall intensity distribution within the DoI rather than individual pixel values. In order to assess the actual potential of the modality in tumor detection, blinded studies have particular relevance.

### 3.3. Microwave Breast Imaging Devices

This section revises the previous analysis conducted by O’Loughlin et al. in [61] by incorporating the latest developments since 2018. This includes a focus on newly published results from clinical experiences and an exploration of the current strategies and focuses of the companies active in this field. Table 1 compares the primary operating systems based on their key technical feature: array type and geometry, antenna type, number of antennas, operating frequency, coupling medium, imaging algorithm, and scan time. We remark that for the array type, two cases are considered:synthetic, where a low number of transmitters and receivers are mechanically moved to complete the surface scan, and hardware, where the array is formed by fixed antennas. Moreover, the summary reports the sensitivity, i.e., the percentage of patients correctly identified as having a tumor, obtained in the largest trial relative to each device. Further details on each device are given in the following.

The first human-tested system was pioneered by Prof. Meaney’s team at Dartmouth College (D-C), Hanover, NH, USA in 2000 [73]. The device is a tomographic scanner characterized by a cylindrical synthetic array of 16 transceivers (movable in the vertical direction). In 2012, the group released an updated version of the 3-D tomographic scanner, including a series of effective hardware innovations to enhance data quality; also, the first large-scale trial in a medical facility with more than 400 exams has been documented [74]. Cross-plane multi-view measurements are collected, employing two interleaved sets of eight antennas separately moved upright, suited to all breast sizes. The breast is immersed in a glycerin–water coupling liquid. The authors note that multi-frequency acquisition allows for flexible resolution adjustment for different breast densities. The image reconstruction takes less than 20 min, employing an original logarithmic version of Gauss–Newton iterative reconstruction [30]. Overall, the system provides a resolution of around 1 cm and a scan time of 2 min per breast. Although no statistical analysis was provided, two relevant sample cases are analyzed: first, it confirms adequate sensitivity in detecting invasive ductal carcinoma of size 1.2 × 1.3 × 1.0 (cm), while comparing counter-image of the healthy breast, almost homogeneous; the second example demonstrates monitoring capability during neoadjuvant chemotherapy (NCT) through six follow-up measurements, using MRI at the beginning and end of the treatment period as the gold standard. In 2013, the system was further tested for monitoring the progression of breast cancer response to NCT in eight patients [75]. The evaluation of the mean conductivity in the DoI was suggested as an effective approach to determine the level of response to the therapy [75]; in particular, at one month after the start of therapy, the value is statistically different between the complete and incomplete (non)-responding patients. To the best of our knowledge, there have been no recent publications of clinical trial results using this system.

The team of UBT s.r.l., Perugia, Italy, built a novel device for breast tumor detection, called MammoWave, that recently received CE mark and ISO certification. It operates using two antennas that rotate azimuthally in air at 10 transmitting positions, while still retaining proper scanning speed [76]. Here, the images are conductivity-weighted dielectric maps obtained via radar approach based on Huygens Principle (HP) [25]. Image feature analysis, based on MW images’ parameters, quantifies non-uniform patterns to distinguish breasts with no radiological findings (NF) from those with findings (WF); both benign and malignant lesions are included, e.g., microcalcifications, cysts, and masses in the order of tens of millimeters. The clinical dataset comprises a balanced group of 103 breasts, of which there are 52 NF and 51 WF, where each group includes also dense breasts. Two methods of features analysis are tested: using each single feature separately, an AUC between 0.65 and 0.69 is obtained; by utilizing an appropriate empirically derived combination of features, the classification achieves a sensitivity of 74% (82% in dense breasts). Recently, the group implemented an ML-based classification approach-based backscattered signals recognition via support vector machine (SVM) with radial basis function [77]. A sensitivity of 84% and specificity of 95% in detecting WF cases is reported on a cohort of 61 patients aged between 20 and 80. We recall that the specificity is the proportion of tumor-free cases accurately identified. It is noted that no image-based evidence is provided; thus, the capability of localizing the actual lesion cannot be evaluated. A large-scale clinical trial on 600 volunteers, the highest number up to date, is currently ongoing in a multi-center collaboration project [78] and aims to assess imaging capabilities for breast lesions detection, extending the investigation in [76]. Among possible limitations of the study, the accuracy in positioning the breast in the cup depends on the medical operator only.

At the University of Calgary, AB, Canada, Prof. Fear and her team developed monostatic UWB radar imaging technology, using the DAS-type algorithm, referred to as the tissue sensing adaptive radar (TSAR) [27,79]. A single antenna is scanned around the breast (with 3-D covering), positioned via laser-aided patient-specific surface recognition [84]. In [79], a prospective investigation on eight suspicious lesions demonstrated the capability of clearly detecting the target area, obtaining consistent response even in the case of three more complex cases of ductal carcinoma, where the sensitivity of MRI is limited. From observation in [79], the possibility emerges of using the intensity threshold and left and right breast image comparison to detect low but significant responses due to tissue inhomogeneity. The experience gained in the academic years also led to the creation of the company Wave View Imaging devoted to the clinical development of fast and convenient imaging technology for breast density assessment, screening, and treatment monitoring [85].

Prof. Fear and colleagues’ research activity also produced the microwave imaging transmission system (MITS), with an innovative design more similar to MRI one, with two five-probe planar arrays lightly compressing the breast opposite sides [29,69]. The differential algorithm implements time delay spectroscopy (TDS) based on two scans at the same separation distance, with and without the breast, resulting in low-resolution 2-D images. Very fast multi-view acquisition allows completing the procedure within 10 min. After consistency validation on phantom [29], a recent pilot study on 15 patients aimed at monitoring early stage breast cancer development after surgery and radiation therapy (at 6 weeks, 1 and 2 years post-treatment), integrating traditional clinical timeline of scans. Based on currently available data at baseline and 6 weeks scan, two fundamental results were reported: consistency of the properties estimate of the untreated breast over time, and a statistically significant increase in permittivity related to the treatment physiological response (e.g., inflammation), which fades in later examinations after surgery in case of positive outcomes [69].

Radar-based MW imaging systems MARIA was developed by Micrima Lt, Bristol, UK, following research work at Bristol University, UK [26,80,81]. It has undergone rapid development through several device generations receiving CE marking; the latest 6th generation trial was announced in 2024. Multi-static data collection is provided through a 60-slot antennas-array electronically switched, fixed in a hemispherical shell which is filled with liquid CM during the measurement. The method suggested to address strong skin reflection in this configuration is based on the signal comparison between two measurements at shifted angles, where only the tumor-related signal varies from the point of view of each antenna pair. Results in [80] show consistent performance in detecting both benign and malignant lesions in breast tissues, considering also dense breasts, with a notable achievement of detecting lesions as small as 5 mm. In a population of 225 patients, the overall sensitivity is 76%, similar in benign and malignant lesions. The trial methodology remarkably includes blind and unblind radiologist assessments, and analyzes influencing factors like menopausal status, breast density, and age groups. According to the study in [81] on 389 patients, the largest to date with MARIA M6, 47% of malignant lesions match with the reconstructed image intensity. The authors observe that the information gathered from the deeper tissue and in the presence of small lesions may be more attenuated. Worth noticing, Micrima Lt is introducing on the market a new handheld, rapid, painless MW-based scanner for breast density analysis [86]. It is devoted to complementing the traditional healthcare pathway by identifying a priori the most suitable imaging technique for detecting cancer based on each patient’s breast tissue type, avoiding useless exams and the costs of secondary tests.

The Wavelia system, developed by MVG Industries, Villejust, France, reported its first in-human clinical investigation in 2021 [70] with the support of the University of Galway, Ireland. Mechanical scanning of the breast is performed in a hemispherical hole with a circular multi-probe array moving vertically at 2 mm steps, aided by optical breast contour detection. Radar imaging is based on the time reversal TR-MUSIC algorithm [22] and data-driven EM propagation speed estimation related to the percentage of fibroglandular tissue. Results from a small-scale investigation (24 patients) in 2022 verified patient tolerance and the feasibility of the imaging reconstruction, with an overall sensitivity of 87.5% among different types of cancerous lesion. The patient is required to lay prone for about 15 min. Wavelia#2 implements notable evolution to the imaging procedure, accelerates the processing and addresses the arrangement of different breast size, stabilization of the matching medium properties, and extension of the sensing volume to the posterior breast, a critical area often shadowed with traditional compression-based configurations [82]. The prototype is currently under testing on approximately 70 patients, while the team in [82] preliminarily assessed the repeatability of tumor detection on realistic heterogeneous breast phantoms, remarking the importance of pilot investigation steps in the validation procedure. The image reliability relative to consecutive scans and exams in different days is promising for future expectations.

Mitos Medikal Technologies A.S., Istanbul, Turkey, and the Istanbul Technical University, Turkey, developed the Scan and Find Early (SAFE) clinical device for breast screening, now under the CE certification process [23,83], a mobile bistatic cylindrical system sampling 36 different angle positions, offering size-adjustable cups, and where matching is given by a solid ceramic medium. The total scanning is performed in about 20 min [23]. A Supervised ML method, namely Stochastic Gradient Descent (SGD) is implemented in the first step to detect the presence of a lesion based on the scattering transmission parameters. For the localization of anomalies, two qualitative 2-D algorithms are employed, i.e., the linear sampling method (LSM) and Factorization Method (FM). In [71], the screening imaging capability is validated on 115 patients, achieving 63% overall sensitivity, negatively affected in small breast size as well as in benign lesions with respect to malignant ones. Sensitivity in lower-density breasts increases up to 86%. The trial methodology involves trained medical staff in non-blinded evaluation and the population presents diverse pathological cases classified as benign, high-risk, or malignant. The smallest detected lesion is 6 mm, while the average mass size is around 26 mm. In 2023, the validation was extended to ML-based lesion classification [72]. In a study involving 113 patients with exclusively high-density breasts, an Adaptive Boosting model is proposed to distinguish backscattered signals in pathological breasts. The model achieves a sensitivity of 79% and specificity of 77%; moreover, SAFE misclassifies 25 out of 113 breasts (False Positive: 16, False Negative: 9), thus achieving an accuracy of 78%. The authors note a slight improvement in performance among younger patients, contrary to traditional mammography. However, this observation requires confirmation in larger studies that consider both age and breast density collectively. The authors state that nowadays, SAFE can detect lesions as small as 3 mm, with clinical testing involving over 1000 patients, showcasing sensitivity and specificity values of 81% and 83%, respectively [83].

Although the technology development seems to favor table-embedded designs, the academic literature also proposes interesting wearable or handheld systems to be placed on patients in the supine position [87,88], whose main focus is breast health monitoring. Cost-effectiveness and portability are desirable features for the application; moreover, no matching medium requirements and ease of breast surface contact appear to improve the quality of collected data [88].

## 4. Brain Stroke Detection

### 4.1. Brain Stroke Diagnosis and Treatment

Brain stroke is a life-threatening disease estimated to affect one in four people in the world’s adult population, with a higher burden in low-to-middle-income countries [89]. Lasting brain damage and disability may follow a stroke event, seriously impacting people’s lives but also representing a significant financial strain on healthcare resources, arousing a growing interest in the medical market for clinical diagnosis and treatment devices. Current medical protocols for stroke care differ based on the stroke type. Approximately 85% of cases are ischemic strokes (IS), caused by a clot obstructing blood and oxygen flow to the brain. The remaining cases are typically intracerebral hemorrhages (ICH), which occur due to blood vessel leakage. Timely and accurate diagnosis followed by swift transportation to a well-equipped medical facility is crucial, with guidelines recommending neuroimaging evaluation using CT or MRI. Initial interventions for ICH focus on managing intracranial blood pressure and eventually emergency surgery [90]. For IS cases with small lesions and patients arriving promptly after symptom onset, treatment may involve tissue plasminogen activator (tPA) to dissolve blood clots, with thrombectomy reserved for severe conditions [91]. Clinicians refer to the “golden hour” following IS onset as a critical period where prompt medical action significantly improves the survival rate and reduces long-term complications [91]. Despite the well-documented clinical relevance, standard imaging methods are plagued by issues such as time-consuming procedures and high costs that limit their availability. Moreover, ongoing debates regard their effectiveness for real-time treatment monitoring and follow-up, particularly when considering the ionizing radiation risks associated with CT scans. These challenges within the diagnostic framework encouraged the emergence of MW imaging as a promising solution.

MW-based devices, characterized by their portability and wearability, integrate advanced yet cost-effective technology capable of fast scanning and data processing. They are suitable for repeated safe irradiation, which may complement current clinical protocols. Despite facing several hurdles and limitations in real-world applications, MW imaging is now recognized as a valid candidate in the market of Mobile Stroke Units (MSUs), specialized prehospital stroke services provided in ambulances to offer immediate diagnoses, particularly in rural and underserved areas [92]. The competition with portable CT and MRI equipment, which have been under assessment since the early 2000s without achieving widespread use, primarily centers around the accuracy-to-cost ratio, a critical consideration from both industry and medical standpoints. Furthermore, it is worth noting that next-generation brain stroke diagnostics is exploring alternative technologies such as electroencephalography (EEG), ultrasonography, and near-infrared spectroscopy (NIRS) [93].

A comprehensive review of MW-based systems for brain stroke imaging is provided in [9] by Guo et al., which details recent progress in hardware and processing algorithms. A crucial challenge in brain image is related to the complexity and inhomogeneity of the head tissues. To address a favorable balance between penetration depth and resolution the optimal frequency range is typically between 0.5–2 GHz [94]. Antenna design encompasses free-space antennas, high permittivity ceramic-loaded rigid waveguide antennas, and on-body matched antennas. Free-space antennas require strategies to mitigate strong reflected signals and may not be suitable for clinical settings; however, some examples are provided, such as the metamaterial-loaded wideband antenna proposed in [95]. Ceramic-loaded waveguide antennas can be bulky due to their weight. On-body matched antennas, usually immersed into a CM to improve penetration and reduce antenna size, face challenges such as unstable phase centers and unidirectional radiation [9].

Both qualitative and quantitative imaging methods are reported in the literature. The most suitable methods for real-time usage are radar-based methods but also ML-based strategies. However, quantitative algorithms are valuable to provide information regarding stroke type and tissue distribution. Differential imaging techniques, such as the approach outlined in [96], offer effective methods for tracking stroke progression over time by monitoring changes in dielectric properties.

### 4.2. Microwave Brain Stroke Imaging Devices

The academic community made a major contribution to the MW technology development, giving rise to the first measurement prototypes, which explore a broad universe of hardware and software solutions, depending mainly on the target application. At this level, the preliminary experimental tests are usually conducted on realistic head phantoms, where the stroke-affected region can be represented with a similar dielectric body, which may eventually expand within the brain [31,97,98,99,100,101]. The remaining of this section reports, first, the more relevant academic devices that comprise both 2-D [31,102,103] and 3-D [96,104] configurations. Then the products realized by companies are detailed. Compared to Section 3, here, we decided to provide additional insights into current academic research, as the application of MWs to brain imaging is significantly more recent compared to its use in breast imaging.

The research team based in Malaysia proposed a remarkable prototype which consists in a portable 9-antennas switched-array system for ICH detection, tested on a realistic multi-tissue phantom in [102]. The antennas work in free space, rotating at different angular position at short-distance from the head. To deal with air–skin interface reflection, the authors incorporated metamaterial in the antenna design to enhance the radiation efficiency. By combining an updated version of the DAS imaging algorithm in [28], 2-D images were promising in ICH early detection.

At the University of Genoa, Italy, an advanced multi-static system harnesses a circular 16-antenna array, and the imaging is addressed through a cutting-edge variable-exponent Lebesgue-space regularization technique [103]. The transceivers feature slotted cavity-backed bowtie antennas, tailored to operate within the frequency spectrum spanning from 500 MHz to 2.5 GHz, and matched to the skin via polyethylene bags filled with glycerin–water CM. The proposed quantitative imaging methodology leverages stepped frequency data, dynamically refining the Lebesgue-space exponents through adaptive adjustments following each inexact Newton iteration. In experimental validation, the prototype demonstrates remarkable efficacy on simplified 2-D scenarios, notably discerning cylindrical inclusions measuring 2 cm and 5.2 cm in diameter within a cylindrical tank.

The research team from King’s College London, UK, developed an experimental prototype for brain stroke detection and classification [31]. Their inversion strategy, implemented via finite difference time domain (FDTD) solver, utilizes the distorted Born iterative method combined with the two-step iterative shrinkage thresholding (DBIM-TwIST) algorithm to address the inherent ill-posedness of the problem. Data acquisition is accomplished by a ring-array of eight spear-shaped antennas deployed on multilayer phantoms with inclusions designed to emulate both ICH and IS conditions. While experimental reconstructions in 3-D scenarios exhibit slightly reduced accuracy compared to 2-D tests, they yield greater difference in dielectric estimation between the ICH and IS targets. Furthermore, in [105], the research is centered on enhancing antenna performance through the incorporation of a metasurface (MTS) impedance-matching layer. The MTS unit cell comprises a metallic lattice based on the Jerusalem Cross, embedded between two high-dielectric substrates. Numerical analysis indicates that the addition of MTS effectively mitigates undesirable reflections at the skin interface and amplifies transmission within the working frequency band 0.5–2.0 GHz used for brain imaging. This augmentation widens the distribution of the E-field, resulting in improved signal coverage within the brain region. Experimental investigations conducted on a simplified homogeneous head phantom illustrate that both tomographic DBIM-TwIST reconstructions and radar-based imaging exhibit superior accuracy in target localization and reduced artifacts with the incorporation of MTS.

Recently, the research group from the University of Campania “Luigi Vanvitelli”, Italy, reported advancements in a compact helmet-hold device called TES (subcranial ENcephalic Temnograph) for real-time stroke monitoring [104]. This device employs a 16-element array of miniaturized slot antennas operating in the 1–2 GHz frequency range. Each antenna is individually controlled by a pneumatic mechanical system of micro-pistons that applies a precise mechanical force to ensure proper contact with the skin and prevent antenna movement. The MW system exploits differential scattering measurements (at different time intervals) and the Incoherent MUSIC algorithm; 3-D image reconstruction is achieved by a slice approach as a collection of 2-D pseudospectrums. Aiming to improve portability and cost-effectiveness, the team integrated a custom RF circuit for multi-view multi-static matrix data collection, enabling head scanning within four minutes. Experimental validation conducted on a four-tissue head phantom showcases the device’s capability to detect hemorrhagic inclusions as small as 16 mL in volume.

The Wavision research group from Politecnico di Torino, Italy, and the Institute for the Electromagnetic Sensing of the Environment, National Research Council of Italy (IREA-CNR) proposed a low-complexity scanner intended for brain stroke monitoring in acute and post-acute phases. The first generation dates to 2020 [106], then newer upgrades toward wearable and portable structure are testified in [96]. A 3-D array of 22 monopole antennas is matched to the skin surface by a semi-flexible brick, connected to a 2-port VNA via ad hoc electromechanical switching [96]. The entire acquisition is completed in about six minutes, then a differential linear imaging algorithm, namely, the Truncated Singular Value Decomposition (TSVD) [24], estimates in a few seconds the 3-D map of the dielectric contrast variations. The apparatus was first tested on a homogeneous antropomorphic head phantom, demonstrating the ability to localize and track mimicked stroke evolution in both hemorrhagic and ischemic lesions [96], then further validation on custom multi-tissue dynamic phantom was performed in [97,107]. Currently, the team effort is also headed to raise the device compactness together with measurement robustness, incorporating an off-the-shelf solid-state switching matrix [108].

Collaborative efforts among academia, industry, and medical institutions have culminated in creating the first physical systems authorized for human testing. As far as we know, three primary active devices are undergoing trials on patients. A detailed description is provided below, and Table 2 compares their main characteristics. It is noted that only one clinical trial in the table reports the value of accuracy achieved, that is, the percentage of correct classifications among all the cases.

Medfield Diagnostics AB, Göteborg, Sweden, founded after promising research at Chalmers University of Technology, Göteborg, Sweden [116,117], developed a tool for prehospital screening and triage support in the emergency department, to be used in the ambulance or at the patient bed, in case of suspected stroke or traumatic brain injury. In the preliminary publication in [116] (2014), the team developed the first system prototype with optimized radiating array designs usable in the clinic. Supervised learning and a detection algorithm based on the subspace classifier “CLAFIC” are employed. Two proof-of-concept studies, on 20 and 25 patients, respectively, demonstrated promising performance, for example, in the second case, a detection sensitivity of 90% was achieved for HS with a specificity of 65% [116]. The latest device version is the Strokefinder MD 100, designed for the classification of cerebral hemorrhage and ischemic stroke, both in the acute phase and during thrombolytic treatment, and monitoring the early stage brain evolution [109]. It comprises eight antennas arranged in four pairs, encircling the patient’s head ensuring optimal contact, and the measurement takes around 45 s [118]. In 2017, a clinical study tested the Strokefinder MD 100 measurements combined with a diagnostic classification algorithm for the differentiation of traumatic ICH patients and healthy control subjects [118]. The exploratory subset comprised 20 ICH cases, patients admitted for surgery for chronic subdural hematomas of large size (mean volume of 112 mL), and 20 healthy control cases. The analysis achieved 100% sensitivity and 75% specificity, i.e., all hematomas were detected at the cost of 25% false positives. These results indicate the viability of the method for early clinical diagnosis of traumatic brain injuries, although further investigation is needed for acute ICH, which may have different dielectric characteristics and smaller sizes. In 2024, a research team from Greece published the outcomes of a feasibility study for bedside use of the Strokefinder MD 100 within realistic clinical protocols [119]. The study aimed to evaluate the possibility of MW-based adjunctive support to patient emergency care with no additional time with respect to the traditional clinical path. A total number of 71 patients with suspected stroke after triage were recruited, and for almost 90% of this cohort, a useful result was provided within 10 min, while the remaining failed due to system or user errors. Moreover, the authors reported positive feedback from the medical staff. Currently, Medfield is conducting a pilot study for deploying Strokefinder on ambulances in Australia and is further validating its effectiveness through open multi-center trials aimed at verifying classification on a comprehensive dataset consisting of both pathological and healthy patients [109,120].

EMTensor GmbH, Vienna, Austria, [110] and Dr. Semenov’s team at Keele University, UK, and Carolinas Medical Center, Charlotte, NC, USA, have developed a tomographic system for stroke detection. The company is targeting two main applications, i.e., bedside brain imaging and in-ambulance early triage of stroke. Their second-generation system is described in [121]. The inversion strategy uses FDTD simulated electric fields and applies gradient-based minimization of a real-valued non-negative functional and standard Tikhonov regularization. Experimental validation on phantom showed that the system was clearly able to localize and describe the ICH and surrounding tissues’ dielectric properties. A clinical prototype has been developed by integrating a multiport VNA in a portable, space-saving packaging [112]. The measurement spherical chamber contains 177 ceramic-loaded rectangular waveguide antennas distributed on different rings surrounding the entire head volume, and controlled through a parallel architecture realized with custom-built transceiver Printed Circuit Boards (PCBs) [122]. The experimental study in [122], exploited the EMTensor measuring capabilities combined with an innovative 2-D non-linear inversion strategy applied in Lebesgue space. The actual version EMTensor EMT BRIM G3 scanner underwent the first pilot feasibility clinical study on 10 healthy volunteers and 30 real stroke patients, approved by the Ethical Committee of Upper Austria. Consecutive days exams provided a total of 52 scans to analyze and compare with CT or MRI scans as reference. The prompt use of the scanner on early stroke phase is guaranteed by the fast acquisition time, less than 2.5 s for the operational frequency band 0.92–1.08 GHz, and the compact wheeled device cart is easily moved to the patient bed. The authors reported positive concordance with the gold standard, promising potential in detecting and differentiate the lesion nature [113]. According to the latest updates to the system reported in [32], the array has been reduced to 128 elements arranged in 4 rings, reducing the overall power consumption. To ensure real-time image reconstruction, the company exploits a cloud platform for extensive computational resources and advanced deep neural network techniques, which are expected to improve the performance accuracy as the ongoing clinical studies expand the data storage.

The brain scanner by EMVision, Sydney, Australia, is the product of over a decade of research and development at The University of Queensland, Brisbane, Australia [111]. The technology developed in academia has been exploited to investigate several types of radar, tomographic, or ML-based algorithms [115]. In addition, an alternative strategy in [123] is based on a comparison of signals crossing lines while assuming symmetric behavior of the 2-D imaging scenario. The simple, fast procedure does not require a priori information of the healthy scenario, aiming to overcome common limitations in MWI such as lack of knowledge of the internal tissue, variability among subjects, and computational burden of some tomographic algorithm. Statistically significant experimental testing on phantoms certified the possibility of accurately estimating ICH location and size, fostering further investigation on 3-D implementations and other injuries [123]. The researchers explored a 3-D flexible antenna array wearable as a cup in contact with the head, tested for radar-based imaging on realistic phantom [124]. The EMVision commercial prototype features a portable ultra light weight standalone headset to operate in the critical phases of stroke care, i.e., pre-hospital diagnosis, support of clinical intervention, and bedside monitoring. A recent report published in [115] details the main technological solution pursued in the second version of the clinical prototype. The antenna array consists of a single ring of 16 waveguide-type radiators which covers a slice of around 60 mm in the head as the imaging domain. A thin silicon membrane contains the liquid matching medium, and each transceiver is connected to a different port of a VNA, scanning a frequency band between 0.7 and 1.8 GHz. The need for offline and in-line calibration is addressed by implementing specific solutions involving homogeneous calibration phantoms and differential signal analysis among consecutive measurements. The processing software completes consecutive tasks accomplished by different algorithms, including reflection coefficient-based boundary detection, localization and classification of ICH and IS strokes from mapping of the scattering signal to graphs, beamforming combined with tomography and unsupervised ML for target focusing, and finally, the combined output of complementary algorithms is determined by the overlapping region of agreement. Overall, the processing takes around 1 min, allowing quasi-real-time implementation. A trial conducted on 50 patients (37 ischemic and 13 hemorrhagic) at the Princess Alexandra Hospital in Brisbane, Australia, demonstrated the ability to localize the injured brain area with 80% accuracy (in two-dimensional quadrant localization), and an overall accuracy of 98% in stroke type differentiation.

## 5. Non-Invasive Glucose Monitoring

### 5.1. Diabetes and Blood Glucose Level Sensing: State of the Art

Diabetes is a chronic metabolic disorder marked by abnormal blood glucose levels (BGLs), resulting from either insufficient insulin production or the ineffective use of insulin by the body. Long-term effects involve significant damage to various body systems, particularly the nerves and blood vessels. According to the WHO, mortality rates from diabetes have remained persistently high over the past two decades, with a notable increase in lower-middle-income countries [125]. The normal fasting BGL range is 70–100 mg/dL, but in diabetic patients, levels can exceed 200 mg/dL, requiring immediate medical attention.

Nowadays, the gold standard for measuring blood glucose is the invasive evaluation of a drop of blood from the fingertip. Despite its high sensitivity and accuracy, this method has significant drawbacks, including pain and risk of infections. As a result, alternative solutions using minimally invasive or non-invasive sensors have been explored, with some receiving commercialization authorization [126]. The application of greatest interest is Continuous Glucose Monitoring (CGM), which is highly beneficial for self-adjusting insulin dosage and also holds significant value for clinical applications [127]. The most prevalent CGM technique is based on glucose-oxidase electrochemical sensors [128]. However, these sensors have drawbacks such as their limited lifespan (ranging from days to months), a non-linear response within the biological range, and performance dependence on enzyme availability on the electrode surface. Additionally, there is a response delay of 5 to 10 min due to the lag between sensed glucose concentration in the interstitial fluid and the concentration in the blood, which is crucial for real-time decision-making [128]. To evaluate the suitability of a CGM system, accuracy is a crucial parameter assessed using various metrics. One such method is the mean absolute relative difference (MARD), which represents the relative difference between the predicted data $d_{P}^{\left(i\right)}$ and the ground truth (GT) $d_{GT}^{\left(i\right)}$, obtained from standard procedures across a dataset of *N* measurements:

(2) $$MARD=\frac{1}{N}\sum_{i=1}^{N}\frac{|d_{P}^{\left(i\right)}-d_{GT}^{\left(i\right)}|}{d_{GT}^{\left(i\right)}}.$$

**Figure 3:**
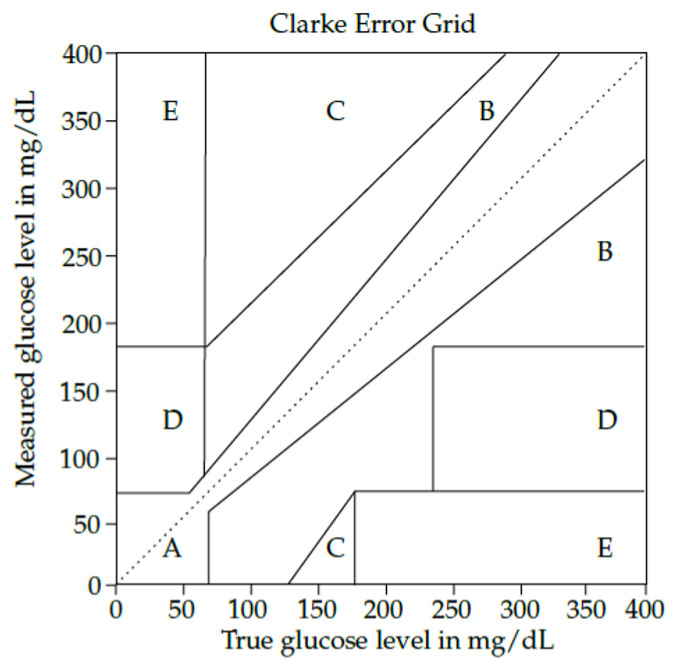
Clarke error grid analysis for glucose monitoring devices. Regions A and B correspond to clinically valid treatment and uncritical treatment, respectively. Outside these areas, the detection is considered clinically not acceptable. Image from [130].

Specific consensus criteria define the clinical acceptance range for CGM devices based on error grids, such as the Clarke error grid (CEG) [129], shown in Figure 3. CEG regions have the following meanings:

- Region A: measurements within 20% of the reference sensor, corresponding to clinically valid treatment;
- Region B: values outside of 20% but not leading to inappropriate treatment;
- Region C: measurements that result in unnecessary treatment;
- Region D: measurements indicating dangerous failures to detect and treat;
- Region E: “erroneous treatment” zone, where measurements cause confusion between the treatment of hypoglycemia and hyperglycemia.

Therefore, a diagnosis and treatment are considered clinically acceptable only if the value falls within regions A and B.

The next generation of CGM devices is focused on EM-based sensing of blood dielectric properties, which vary with glucose concentration. Techniques span the EM spectrum and include impedance spectroscopy (radio waves), and MW sensing up to optical sensing (nm waves) that still allows sufficient penetration to examine blood vessels beneath the skin [130].

**Figure 4:**
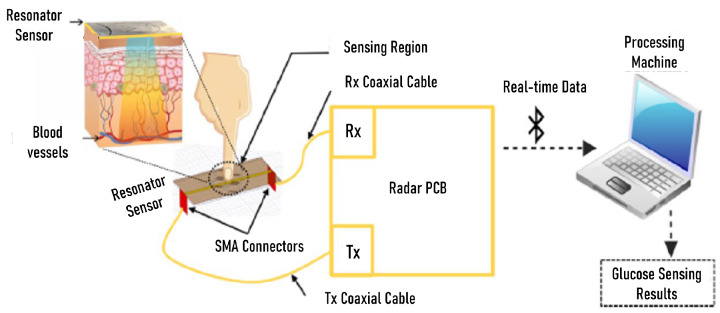
Scheme of the portable MW sensing system in [139].

MW-based technology leverages the sensitivity of scattering parameters, namely, reflection coefficients (S11) and transmission coefficients (S21), to changes in blood permittivity that are dependent on glucose levels. Two possible variables are observed: (1) the magnitude of scattering parameters, and (2) the resonant frequency. Different estimation methods can then be applied to determine the glucose level, such as model fitting [131] or more complex ML-aided regression analysis [132,133]. For example, some studies exploit the linear relationship between resonant frequency shifts and glucose concentration [131,134,135,136]. One significant advantage of MW sensing is better tissue penetration compared to optical frequencies. Additionally, sensors are often fabricated using PCBs, which are cheaper than optical sensors. However, the primary cost in current experimental systems stems from VNA-based stimulus signal generation; thus, to make this technology viable for affordable CGM devices for home use, alternative hardware solutions have been developed in [137,138]. The radar-driven sensing system in [139] is an example of a portable, cost-effective, and low-power solution (the working principle is illustrated in Figure 4).

One main challenge common to wearable monitoring applications is dealing with body movements, which may lead to inconsistent results. For this reason, it is crucial to stabilize the body area interfacing the sensor and implement specific designs immune to movements and bending losses. Furthermore, the sensor must have high specificity for glucose level variation rather than other similar compounds in the blood (e.g., fructose and galactose). Highly sensitive sensors, such as split-ring resonators (SRR) and patches, are commonly used because of their capability to offer localized regions sensitive to biological capacitive variations [132]. Combining two or more designs can enhance the sensitivity and reduce the sensor’s size as demonstrated in [132,136,139,140]. The proposed spoof surface plasmon polariton (SSPP) endfire sensor in [135] has low effective aperture and includes triangular ground planes to suppress the sidelobes, resulting in a slow-wave characteristic. Ref. [136] proposes a contact-based meander-line antenna sensor (CMS), where the combination of a meander-shaped design with another structure aims to confine the fields, reducing the leaky losses. According to [131,141], a high-quality factor and highly concentrated fields in the sensing area improve the sensitivity. A whispering gallery resonator (WGR) is adopted in [141] to amplify the dependency between the signal amplitude and glucose levels. Finally, effective solutions to enhance robustness against noise and EM interference can be non-reciprocal measurement [142] and dual frequency operation [131,133].

### 5.2. Microwave Sensors for Glucose Monitoring Tested on Humans

In this section, we report a more detailed description of the latest MW sensors tested on humans (from 2020 onwards). We build on the reviews found in [130,137] and include some novel products [132,133,138,140]. The selected systems are compared in Table 3, which includes the main publication reference; the sensor type; the operational frequency in GHz, which is a single resonance frequency, two values for dual-frequency applications, or a frequency range; the observed sensed variables, that can be the amplitude and the resonance frequency shift of S11 and S21 parameters; the range of glucose levels in mg/dL tested in the measurements; the method employed to estimate glucose levels based on the sensed variable; the number of subjects included in the trial population, categorized as diabetic (D), non-diabetic (ND), or pre-diabetic (PD); the sensitivity, quantified as the variation of the sensed variable (i.e., frequency shift, in MHz, or amplitude variation, in dB) with a small change in glucose concentrations (1 mg/dL); and the accuracy, evaluated using the MARD value according to Equation (2). Most sensors are designed for fingertip placement [131,133,134,138,140], while two studies are tailored for arm sensing [135,136] and another for pancreas sensing [140]. Usually, preliminary trials consist of controlled monitoring during glucose tolerance tests lasting a few hours, with subjects ingesting glucose following fasting periods. Standard devices for BGL measurement are employed to assess the accuracy. Despite the studies being limited to small test populations and controlled conditions (e.g., stable temperature and restrained physical movements), the accuracy achieved is comparable to, or also superior to, that of the existing commercial CGM devices [128]. Furthermore, some studies reported CEG analysis with 100% of predictions falling within the clinically acceptable regions [132,133,134].

The novel devices not described in previous reviews are detailed below. Ref. [138] presents an embedded fingertip measuring system for non-invasive CGM using an RF one-port transceiver made of off-the-shelf elements. The system measures S11 and observes the resonance frequency to extract a relationship with the glucose levels. The human test is performed first on 43 healthy volunteers detecting glycemia five times at time intervals of 15 min. In 80% of the cases, a negative correlation is detected (according to Pearson’s metric), demonstrating the feasibility of the method, although further investigation before deriving a universal relationship is still needed. Furthermore, day-long monitoring is tested with a diabetic patient, where glycemia levels vary approximately between 90 and 190 mg/dL. The value of MARD for this patient is 7.8%, comparable with other commercial systems.

The work in [140] aims to design a home-diagnostic system embedded into a wearable belt for sensing the pancreas zone, whose properties change during insulin secretion. It measures the return loss at 4.20 GHz of an H-shaped patch antenna with an I-shaped slot (HSIS). The integrated MW radar IC and RF transmitter substitute the VNA for a wearable system feasible for measurement during and after various daily activities. An ML-based linear regression algorithm predicts the glucose level, in particular, two different equations are retrieved on data before and after food. In the human tests, a dataset from 150 diabetic subjects is used for regression analysis, and a new set of 30 diabetic subjects is used for the test, reaching 91.85% accuracy on the GT value from a commercial glucometer.

The device in [133] uses VNA signal generation and exploits two miniaturized UWB antennas transmitting through the fingertip. A dual-band strategy merges low- and high-frequency information from two working bands centered at 3.67 and 8.35 GHz, respectively. The BGL intelligent monitoring is given through convolutional neural network (CNN)-based features extraction and non-linear regression network, namely, the long short-term memory (LSTM-R). Here, the input data, namely S11 and S21 magnitudes, are normalized with the scattering parameters of water solution. Worthy of mention is the antenna’s selective response to glucose being verified in comparison to other blood substances in experiments with pig serum solution. Moreover, validation confirms the device’s robustness to environmental interference, such as long-term evolution (LTE) and Wi-Fi signals. This was anticipated, given the higher transmit power of the proposed device (0 dBm). The human trial involves ten healthy volunteers subjected to 2 h oral glucose tolerance tests, repeated three times. Each test provides a dataset of 25 measurements, partially used as a training set. The estimation achieves high accuracy as demonstrated by a low MARD value (5.9%); moreover, all the predictions belong to the clinically acceptable area of CEG. As the training and test sets are derived from the same dataset, the relevance of validation is somewhat constrained. However, the promising outcomes warrant further investigation.

Another example of ML-enhanced glucose sensing is provided in [132]. The device is a compact resonator sensor that exhibits a high linear correlation between its measured response, i.e., the S11 resonance magnitude, and the interstitial glucose levels. The ML algorithmic component detects sensor anomalies and employs LSTM networks to predict glucose-level variations, of great interest for real-time response to threatening conditions in diabetic patients. The sensor design combines an SRR and a loading patch, printed on a flexible substrate with copper shielding for EM interference protection, featuring a highly sensitive resonance around 3.6 GHz. The sensor’s selectivity to glucose concentration rather than additional interstitial fluid ingredients is verified in liquid solutions. Human trials involve two healthy subjects and two diabetic patients, monitored over eight days for three hours each day, collecting 16,000 data points for each patient. Prior to each acquisition, calibration is conducted to align the data with that of the reference commercial device measurement. All recordings fall within the clinically acceptable CEG zones. Additionally, the MARD and root mean square error (RMSE) values range from 3.62% to 6.21%, and from 5.17 to 7.78, respectively, across the four tested people.

As far as we know, currently, no MW-based glucose monitoring devices have received FDA authorization. However, some companies are working on developing commercial solutions, such as the smartwatch-embedded system by Afon Technology, Caldicot, UK [143], and the ML-aided non-invasive device by Know Labs, Seattle, WA, USA [144].

## 6. Other Medical Applications

MW sensing technology holds promise for several other medical applications of clinical relevance, at a more or less advanced stage of development. Notable examples include diagnostics for skin cancer [145,146], and colorectal polyps [147], the detection of axillary lymph nodes in breast cancer patients [8] and knee injuries [10]. Additionally, it has applications in monitoring lung and cardiovascular health [11,146,148], providing image-based guidance for thermal treatments [149,150], and body implant (e.g., devices and prostheses) sensing [151]. Despite ongoing research efforts, experimental tests for many of these applications are largely limited to preliminary trials on simplified phantoms. However, significant review work has been conducted, compiling the results achieved so far, highlighting challenges and limitations, and proposing potential solutions. The following overview, based on previous review papers, outlines some of the most promising applications.

Thermal therapy is widely used for treating various types of tumors and includes two main applications. Hyperthermia aims to enhance the efficacy of chemotherapy and radiotherapy by slightly raising the temperature (4–5 °C), which promotes vascular perfusion [152]. Thermal ablation, on the other hand, induces tumor coagulation necrosis by applying controlled high temperatures, up to 60 °C, offering several advantages over more invasive surgical excision [153]. RF radiation and US are typical heating sources, which can be applied externally for hyperthermia or through thin applicators inserted into the body for ablation.

The use of image-guided intervention has been proven to drastically increase the efficacy and safety of both treatments, i.e., hypertermia and thermal ablation, providing reliable real-time monitoring of heat distribution within the treated area and its surroundings, which is crucial for concentrating the temperature increase on the tumor while preventing damage to nearby healthy tissues. Despite extensive research into various imaging strategies, an optimal solution has yet to be widely accepted [154]. MW imaging has recently emerged as a promising candidate due to its EM compatibility with heating sources, real-time capabilities, compact equipment and safe low-power radiation viable for prolonged sessions. As discussed in Section 2, MW imaging relies on the temperature-dependent EM properties of tissues, which are influenced by water content, even at extremely high temperatures [155]. An initial proof-of-concept study demonstrated the feasibility of MW imaging for thermal therapy through a 3-D simplified numerical test [156]. This was further validated by experiments on bovine samples, which preliminarily assessed the capability of the real-time detection of contrasts between ablated and healthy regions using a single moving antenna measuring the reflection parameters and qualitative differential image reconstruction based on the Born approximation and TSVD algorithm [157]. Key observations included potential secondary effects such as tissue deformation after ablation and interference from the thermal applicator affecting the reconstruction. To enhance data quality, optimizing antenna design is crucial. Slot-loaded antipodal Vivaldi antennas are designed in [158] to operate between 600 MHz and 3 GHz, offering a compact design and limited aperture size suitable for custom arrays that cover limited body areas, such as those used in liver measurement. A complete system featuring an array of eight Vivaldi antennas is designed for monitoring liver ablation, with in silico assessments detailed in [149]. Ref. [150] describes the use of UWB radar imaging in ablation therapy. Experiments with liquid phantoms and three-dimensional DAS beamforming algorithms demonstrate the ability to estimate quantitative temperature-induced changes in dielectric properties, based on a priori database of tissue characteristics. A recent study in [159] assesses an innovative strategy for combining in the same system “heating mode”, where power is supplied to the antennas, and “monitoring mode”, where a network analyzer measures broadband transmission coefficients (S21). Ex vivo MW ablation is performed on bovine liver with two 2.45 GHz directional antennas. Analysis of transient S21 spectra aims to predict ablation zones, which are then compared to ground truth images. A linear regression model is developed to map and predict ablation extents.

The deep penetration capabilities of MWs are attractive for diagnosing thoracic diseases, including heart complications, steatotic liver, and pulmonary edema (water accumulation in the lungs), which in turn is linked to further disorders, including COVID-19-related complications [11,148]. Research on torso MW scanning has been systematically revised in [11]. The state-of-the-art encompasses different sensing array configurations, from linear bed-embedded architectures to circular arrays surrounding the upper body, air-matched on body-matched, even in wearable applications. Antenna design targets low-frequency operation within the optimal working range between 0.5 and 1.5 GHz, compactness, and unidirectional behavior to ensure better image quality. For instance, the innovative compact UWB cavity-backed dual-polarized antenna proposed in [160] is tested on a torso phantom for centimetric-scale water inclusion imaging, demonstrating improved signal penetration, hence a significant enhancement in the signal-to-mean-clutter ratio (SMCR) in confocal image reconstructions. Additionally, ref. [11] highlights that, beyond established radar and tomographic reconstruction techniques, research is advancing in classification strategies based on ML and multivariate energy statistics methodologies, which leverage the symmetry of the body to improve diagnostic accuracy.

Further studies have investigated MW-based respiratory monitoring, which is crucial in intensive care units. In 2024, ref. [161] presented the first on-human experiment of respiration tracking via MW wearable tomographic scanning of the lungs. The system employs 16 compact antennas fixed on a belt worn around the torso. A ML-based Supervised Descent Method supports quantitative image reconstruction, reducing computing demands. The remarkable results are expected to further improve with the integration of more complex EM torso models into the network training and inversion algorithm.

An emerging research branch is dedicated to EM knee imaging, offering an affordable, low-complexity and non-invasive alternative for early diagnosis or monitoring of knee injuries (e.g., ligament tears and cartilage damage) that are prevalent among both the youth and old population, with significant economic impact. Ref. [10] collects preliminary on-phantom experimental studies on knee imaging applications, outlining system requirements and challenges to be addressed to advance MW technology toward clinical translation. Despite the ability of MW methods to image soft tissue, the complexity of the knee junction structure and its mobility pose significant challenges. The delay-multiply-and-sum (DMAS) algorithm offers advantages in avoiding the poor injury detection capability and clutter typical of traditional radar methods. At the same time, tomography has not yet been used for knee imaging, despite its widespread validation in other medical applications. Future developments in both equipment and processing algorithms are expected to address these challenges and ultimately establish MW imaging as a competitive solution for diagnosing and preventing knee injuries.

MW technology has been proposed as an alternative to traditional colonoscopy for the detecting of colorectal cancer precursors or polyps. Traditional colonoscopy uses cameras that have a limited field of view, leading to a high chance of undetected abnormalities. The MiWEndo prototype, for instance, integrates MW imaging procedures (specifically, a modified monofocusing algorithm) into traditional colonscopy. The prototype exploits a cylindrical ring-shaped switchable array of miniaturized antennas that provide transmission parameters at 7.6 GHz, enabling the detection of increased dielectric properties associated with high-grade malignant polyps. An experimental study on fifteen ex vivo human colon specimens achieved an overall sensitivity of 100% and specificity of 87.43% [147].

## 7. Conclusions and Perspectives

This paper has provided an in-depth review of state-of-the-art MW imaging and sensing systems for medical purposes, highlighting both relevant advancements and persistent limitations across various applications. MW imaging has shown promising outcomes in breast cancer screening and brain stroke diagnosis and monitoring, with encouraging results emerging from clinical testing of the commercial products. Ongoing large-scale clinical trials may shed some light on the actual potential of existing MW devices in the near future. Moreover, the principle of MW sensing is appealing for continuous glucose level monitoring, offering a cost-effective and non-invasive alternative to current commercial systems. Further advancements in research are anticipated to enhance a variety of recent applications, with the promising expectation of leveraging MW advantages to complement their current medical potential.

Amid the dynamic evolution of MW technology, the research community suggests several pivotal trends and recommendations to drive future advancements. Firstly, there is a critical need to conduct systematic review and organize a comprehensive database of biological tissue dielectric properties according to standardized guidelines, as existing data may be susceptible to measurement inaccuracies and overlook confounding factors. Moreover, the growing computational capacity and the emergence of quantum computers offer promising opportunities to develop highly precise EM models and enhance solving capabilities, that is crucial for quantitative tomography [2]. Several innovative strategies show promise for improving imaging performance. Examples include multi-modality functionalities [162,163,164] and the integration of a priori information from traditional imaging modalities [17]. ML and deep learning techniques are being actively investigated for image reconstruction, signal processing, and pathological status classification. These approaches aim to fully exploit their potential as more clinical data become available. Moreover, ref. [2] emphasizes the importance of leveraging advancements in wireless communications technology and industrial sensing systems to enhance data acquisition, reducing losses, mutual coupling, and sensitivity limitations.

In conclusion, this paper clearly demonstrates, if it was necessary, that MW technology for medical detection applications can now be considered firmly committed to a translation process. Indeed, advanced operational prototypes, developed from spin-off companies stemming the academic research effort, have been increasingly engaged in clinical assessments. Their recent promising results should enable to efficiently feed the unavoidable technology–clinical “push–pull” required for introducing any new emerging sensing modality in a medical radiology landscape already occupied by well-established and, incidentally, constantly improving operative modalities. Such collaborative dialogue will be essential to identify and focus on real medical needs and to achieve direct engagement with medical staff for informed decision-making and effective implementation at an acceptable overall healthcare cost [165].

## Figures and Tables

**Table 1:** Breast microwave imaging devices tested on human subjects. Images from [26,61,67,68,69,70,71,72]. The listed algorithms are classified in Figure 2.

	D-C [73,74,75] 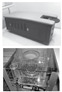	MammoWave [76,77,78] 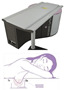	TSAR [27,79] 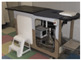	MITS [29,69] 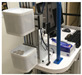	MARIA [26,80,81] 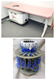	Wavelia [70,82] 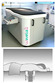	SAFE [23,71,83] 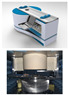
Array type	synthetic	synthetic	synthetic	hardware	hardware	synthetic	synthetic
Geometry	cylindrical	cylindrical	conformal	planar	hemispherical	cylindrical	cylindrical
Antenna	monopole	horn	Vivaldi	Vivaldi	slot	Vivaldi	Vivaldi
No. of antennas	16	2	1	10	60	21	2
Frequency (GHz)	0.7–1.7	1–9	2.4–15	0.1–10	3–10	0.8–4	1–8
Coupling medium	liquid	no	liquid	no	shell + liquid	creamy liquid	shell
Algorithm	tomography	HP	DAS	TDS	DAS	TR-MUSIC	LSM + FM
Scan-time (min)	2	7	30	0.25	0.17	15	7
Largest trial	400	103	8	15	389	24	115
Sensitivity (%)	-	74	-	-	47	87	63

**Table 2:** Brain microwave imaging devices tested on human subjects. Images from [109,110,111]. The listed algorithms are classified in Figure 2.

	Strokefinder MD100 [73,74,75] 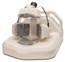	EMTensor [32,112,113,114] 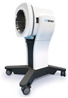	EMVision [115] 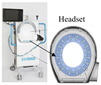
Geometry	conformal	hemispherical	ring
Antenna	patch	waveguide	waveguide
No. of antennas	8	128	16
Frequency (GHz)	0.1–1.95	0.92–1.08	0.7–1.8
Coupling medium	no	gel	liquid
Classification (C)/Imaging (I)	C	I	C/I
Algorithm	ML (CLAFIC)	gradient-based iterative algorithm	custom data-driven algorithm
Scan-time (s)	45	2.5	-
Largest Trial	71	52	50
Accuracy (%)	-	-	98 (C)

**Table 3:** Glucose level monitoring devices tested on human subjects.

Ref.	Sensor Type	Frequency (GHz)	Sensed Variables	Detection Range (mg/dL)	Estimation Method	Trial Population	Sensitivity	MARD
[131]	two cross- shaped resonator	5.5, 8.5	S21 freq. shift	89–262	linear interpolation	11 ND	3.53, 3.58 MHz/(mg/dL)	3% (N = 6)
[134]	microstrip antenna	1.3	S11 freq. shift	60–400	linear regression	75 ND, 50 PD, 125 D	11.4 MHz/(mg/dL)	4.20% (N = 125)
[135]	SSPP endfire antenna	8–12	S11 freq. shift	75–150	not applied	5 ND	3.3 MHz/(mg/dL)	-
[136]	CMS	4.5–5	S11 amplitude + freq. shift	50–280	not applied	5 ND	1.49 MHz/(mg/dL) 0.073 dB/(mg/dL)	-
[138]	not available	1.8–2.2	S11 freq. shift	100–250	3rd order regression	43 ND, 1 D	not available	7.8% (N = 107)
[140]	HSIS antenna	4.2	S11 amplitude	149–290	linear regression	30 D	0.056 (before) 0.027 (after) * db/(mg/dL)	7.34% (N = 410)
[133]	two monopole slot antennas	3.67, 8.35	S11, S21 amplitude	20–500	feature extr. + non-linear regression	10 ND	0.0072 dB/(mg/dL)	5.9% (N = 750)
[132]	patch + split ring resonator	3.6	S11 amplitude	0–500	linear regression	2 ND, 2 D	0.02 db/(mg/dL)	3.62–6.21% (N = 3000)

* Two different regression equations are retrieved before and after food intake.

## Abbreviations

The following abbreviations are used in this manuscript:

AUC	Area Under the Curve
BGL	Blood Glucose Level
CEG	Clarke Error Grid
CGM	Continuous Glucose Monitoring
CM	Coupling Medium
CMS	Contact-based Meander-line Sensor
CT	Computed Tomography
DAS	Delay-and-Sum
DBIM	Distorted Born Iterative Method
DoI	Domain of Interest
DPs	Dielectric Properties
EM	Electromagnetic
FDTD	Finite Difference Time Domain
GT	Ground Truth
ICH	Intracerebral Hemorrhages
IS	Ischemic Strokes
LSTM	Long Short-Term Memory
MARD	Mean Absolute Relative Difference
ML	Machine Learning
MRI	Magnetic Resonance Imaging
MW	Microwave
NCT	Neoadjuvant Chemotherapy
PCB	Printed Circuit Board
RMSE	Root Mean Square Error
SRR	Split-Ring Resonators
SSPP	Spoof Surface Plasmon Polariton
TDS	Time Domain Spectroscopy
TSVD	Truncated Singular Value Decomposition
TwIST	Two-step Iterative Shrinkage Thresholding
US	Ultrasound
UWB	Ultra-wideband
VNA	Vector Network Analyzer
WGR	Whispering Gallery Resonator
WHO	World Health Organization
